# A Simple Risk Stratification Model for ST-Elevation Myocardial Infarction (STEMI) from the Combination of Blood Examination Variables: Acute Myocardial Infarction-Kyoto Multi-Center Risk Study Group

**DOI:** 10.1371/journal.pone.0166391

**Published:** 2016-11-11

**Authors:** Kenji Yanishi, Takeshi Nakamura, Naohiko Nakanishi, Isao Yokota, Kan Zen, Tetsuhiro Yamano, Hirokazu Shiraishi, Takeshi Shirayama, Jun Shiraishi, Takahisa Sawada, Yoshio Kohno, Makoto Kitamura, Keizo Furukawa, Satoaki Matoba

**Affiliations:** 1 Department of Cardiovascular Medicine, Graduate School of Medical Science, Kyoto Prefectural University of Medicine, Kyoto, Japan; 2 Department of Biostatistics, Graduate School of Medical Science, Kyoto Prefectural University of Medicine, Kyoto, Japan; 3 Department of Cardiology, Kyoto First Red Cross Hospital, Kyoto, Japan; 4 Department of Cardiology, Kyoto Second Red Cross Hospital, Kyoto, Japan; 5 Department of Cardiology, Tanabe Central Hospital, Kyoto, Japan; Medstar Washington Hospital Center, UNITED STATES

## Abstract

**Background:**

Many mortality risk scoring tools exist among patients with ST-elevation Myocardial Infarction (STEMI). A risk stratification model that evaluates STEMI prognosis more simply and rapidly is preferred in clinical practice.

**Methods and Findings:**

We developed a simple stratification model for blood examination by using the STEMI data of AMI-Kyoto registry in the derivation set (n = 1,060) and assessed its utility for mortality prediction in the validation set (n = 521). We selected five variables that significantly worsen in-hospital mortality: white blood cell count, hemoglobin, C-reactive protein, creatinine, and blood sugar levels at >10,000/μL, <10 g/dL, >1.0 mg/dL, >1.0 mg/dL, and >200 mg/dL, respectively. In the derivation set, each of the five variables significantly worsened in-hospital mortality (p < 0.01). We developed the risk stratification model by combining laboratory variables that were scored based on each beta coefficient obtained using multivariate analysis and divided three laboratory groups. We also found a significant trend in the in-hospital mortality rate for three laboratory groups. Therefore, we assessed the utility of this model in the validation set. The prognostic discriminatory capacity of our laboratory stratification model was comparable to that of the full multivariable model (c-statistic: derivation set vs validation set, 0.81 vs 0.74). In addition, we divided all cases (n = 1,581) into three thrombolysis in myocardial infarction (TIMI) risk index groups based on an In TIME II substudy; the cases were further subdivided based on this laboratory model. The high laboratory group had significantly high in-hospital mortality rate in each TIMI risk index group (trend of in-hospital mortality; p < 0.01).

**Conclusions:**

This laboratory stratification model can predict in-hospital mortality of STEMI simply and rapidly and might be useful for predicting in-hospital mortality of STEMI by further subdividing the TIMI risk index.

## Introduction

Acute myocardial infarction (AMI) is a disease with poor in-hospital prognosis worldwide. ST-Elevation Myocardial Infarction (STEMI) has a worse prognosis. The development of primary percutaneous catheter intervention (PCI) succeeded in decreasing the in-hospital AMI mortality rate [1]. Nonetheless, the severity and mortality of AMI vary according to patient status, and accurate risk stratification is preferred. Rapid and simple risk assessment for each patient can help in selecting appropriate therapeutic interventions and triage [2].

Several risk stratification models have been reported. The thrombolysis in myocardial infarction (TIMI) risk score could accurately predict STEMI outcomes [2]. The TIMI risk index is thought to be useful in the rapid triage of patients with STEMI during hospital transportation [3]. Vital signs are very important factors of AMI for the interventional cardiologist; the TIMI risk index and the TIMI risk score are simple predictors of in-hospital mortality for STEMI. However, vital signs easily fluctuate with the degree of tension, anginal pain, and the presence of arrhythmia. Therefore, vital signs may vary widely over multiple measurements in many cases. We perceived a need to construct a risk score based on a simpler, more objective biochemical blood examination, which is applicable to any clinical situation and can serve as a more absolute indicator.

Therefore, we developed and assessed a simple and easy risk stratification model from the combination of blood examination variables for in-hospital mortality rate prediction of STEMI.

## Methods

### Patient population and study protocol

**Fig 1:**
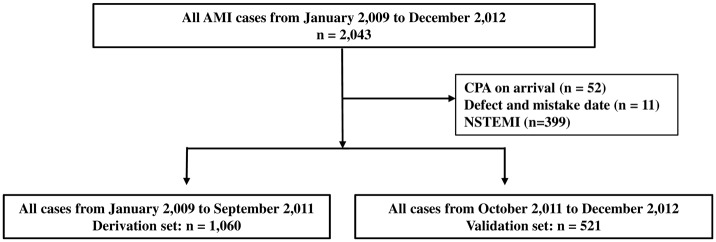
Study protocol and flow chart. AMI, acute myocardial infarction; CPA, cardiopulmonary arrest; NSTEMI, non ST-elevated myocardial infarction.

The AMI-Kyoto multicenter risk study is a prospective, multicenter observational study of patients with AMI who were transferred to 15 participating institutions [4–7]. A total of 2,043 consecutive patients with AMI who were admitted at AMI Kyoto Multi-Center Risk Study Group Hospitals from January 2,009 to December 2,012 were enrolled in the present study. Among these, 52 and 11 patients who had cardiopulmonary arrest upon arrival and erroneous data entry, respectively, were excluded. In addition, 399 patients with Non ST-Elevated Myocardial Infarction (NSTEMI) were excluded. We divided the patients into two groups: derivation and validation sets comprising 1,060 registered cases from January 2,009 to September 2,011 and 521 registered cases from October 2,011 to December 2,012, respectively (derivation set: validation set = 2:1) (Fig 1). The diagnosis of AMI required the presence of two of the following three criteria: (1) characteristic clinical history of ischemic-type chest pain lasting more than 20 min; (2) serial changes on electrocardiogram (ECG), suggesting myocardial infarction (Q waves) or myocardial injury/ischemia (ST-segment elevation); and (3) a transient increase in the creatinine phosphokinase (CPK) and troponin levels to more than 2-fold of the normal laboratory value.

### Data collection

The patients’ demographic information, cardiovascular history, and risk factors (hypertension, hyperlipidemia, diabetes mellitus, smoking, and family history) were recorded. Hyperlipidemia was defined as a total cholesterol level of ≥220mg/dL or the use of cholesterol-lowering agents; hypertension was defined as SBP of ≥140/90 mm Hg or the use of antihypertensive treatment; and diabetes mellitus was defined as a fasting blood sugar (BS) level of ≥126 mg/dL or the use of specific treatment. The SBP at admission was defined as the first SBP recorded in the supine or sitting position upon arrival at the emergency room or out-patient clinic. Based on the WHO criteria, anemia was defined as hemoglobin (Hb) level of <13.0 g/dL and <12 g/dL for men and women, respectively. All in-hospital data were transmitted to the center at the Department of Cardiovascular Medicine in Kyoto Prefectural University School of Medicine for analysis. The study protocol was approved by each hospital’s ethics committee. Because this study corresponds to an observational study to use only existing data from medical records, we performed the verbal informed consent without the agreement document. We indicated record of the informed consent to the electronic medical recoding system. After each institution approved the participation in this study, this study was overall approved by the ethics committee of Kyoto Prefectural University of Medicine as a generalization organization. In addition, this study was approved by the each institutional ethics committee participated in this study (S1 Table).

### Emergent coronary angiography and primary percutaneous coronary intervention

Emergency coronary angiography (CAG) was performed using the standard technique. The coronary flow in the infarct-related artery was graded based on the classification used in the TIMI trial. Coronary stenosis was evaluated based on the American Heart Association classification, and significant coronary artery stenosis was defined as a reduction of at least 75% of the internal diameter in the right and left anterior descending or left circumflex coronary arteries and their major branches, or 50% of the internal diameter in the left main trunk. Multi-vessels as culprit arteries were defined as simultaneous thrombosis of multiple coronary arteries or undetermined culprit artery in the presence of multiple stenotic vessels on the initial CAG. Primary PCI was performed according to the standard techniques, using the appropriate strategy, based on the discretion of the attending physician.

### Selection and cutoff point of five factors from routine blood examination

Anemia, renal failure, hyperglycemia, increased C-reactive protein (CRP) levels, and/or white blood cell (WBC) count were reported as in-hospital prognosis factors for AMI [8–14]. Based on these reports, we selected five factors related to in-hospital mortality from the blood examination variables performed on admission (WBC, Hb, CRP, creatinine, and BS), assessed the spline curve for in-hospital mortality in each of the five items, and defined the cutoff values for each item based on the clinical application (WBC > 10,000/μL, Hb < 10 g/dL, CRP > 1.0 mg/dL, Cr > 1.0 mg/dL, and BS > 200 mg/dL). We evaluated the utility of the risk stratification model from the combination of blood examination variables.

### Statistical analysis

Proportions and continuous variables were expressed as percentages and medians (interquartile ranges, IQRs), respectively. The derivation and validation sets were compared by using the chi-square and unpaired Student’s *t*-tests for discrete and continuous variables, respectively. To define the cutoff values, we assessed the relationship between each of the five factors with in-hospital mortality rate and plotted them by using the spline curve. To avoid the complex modeling, the spline curve was taken as the range between 0 and 30,000 for WBC, between 6 and 20 for Hb, between 0 and 10 for CRP, between 0 and 5 for Cr, and between 0 and 500 for BS (S1 Fig). The odds ratio and 95% confidence intervals (CI) of each blood examination item in the derivation and validation groups were estimated by using multivariate analysis with a logistic regression model. We used SPSS Statistics version 23 (SPSS Institute Inc., Chicago, IL, USA) for all analyses. A *p* value of <0.01 was considered statistically significant.

## Results

### Baseline and lesion characteristics in the derivation and validation sets

The mean age of the patients in the derivation and validation sets was 70.0±12.4 and 70.1±12.9 years, respectively. No significant differences were found in age, sex, cardiovascular risk factors, and family history. Although most patients were transferred to the hospital by ambulance, more than one fourth of patients arrived by self-triage. SBP and HR upon arrival were identified as prognostic factors in the TIMI risk index, which were similar between the derivation and validation sets. Killip classification IV was observed in 9.7% and 12.8% patients in the derivation and validation sets, respectively. No significant differences were found in the severity between the two sets; peak CPK levels were 1,884 and 1,951 mg/dL for the derivation and validation sets, respectively. The laboratory data related to STEMI mortality rate were reported to be similar in the derivation and validation sets (Table 1). Similar baseline characteristic distribution was found in the patients of the two sets.

**Table 1:** Baseline characteristics.

	Derivation set (n = 1,060)	Validation set (n = 521)	p value
Age, years	70.0±12.4	70.1±12.9	0.159
Age ≥ 75 years, n (%)	423 (39.9)	212 (40.7)	0.765
Female, n (%)	307 (29.0)	145 (27.8)	0.640
Hypertension, n (%)	652 (61.7)	291 (56.8)	0.066
Hyperlipidemia, n (%)	432 (40.9)	209 (40.8)	0.985
Diabetes mellitus, n (%)	329 (31.1)	167 (32.6)	0.551
Smoking, n (%)	407 (38.5)	172 (33.6)	0.059
Family history, n (%)	65 (6.1)	35 (6.8)	0.602
Prior MI, n (%)	106 (10.0)	40 (7.9)	0.168
Previous PCI, n (%)	148 (14.1)	76 (14.8)	0.692
Previous CABG, n (%)	14 (1.3)	6 (1.2)	0.790
Previous stroke, n (%)	80 (7.6)	35 (6.8)	0.575
Hemodialysis, n (%)	15 (1.4)	5 (1.0)	0.456
Laboratory date			
WBC > 10,000/μL, n (%)	477 (45.1)	240 (46.1)	0.713
Hb < 10 g/dL, n (%)	60 (5.7)	37 (7.1)	0.272
CRP > 1.0 mg/dL, n (%)	236 (22.5)	122 (24.0)	0.511
Cr > 1.0 mg/dL, n (%)	336 (31.7)	176 (33.8)	0.398
BS > 200 mg/dL, n (%)	302 (28.6)	150 (28.8)	0.927
Arrival			
Walk-in, n (%)	278 (26.4)	115 (22.7)	0.115
Ambulance transport, n (%)	493 (46.9)	265 (52.4)	0.042
In hospital, n (%)	38 (3.6)	17 (3.4)	0.800
Hospital transfer, n (%)	243 (23.1)	109 (21.5)	0.491
Physical examination on arrival			
Systolic BP < 90 mmHg, n (%)	119 (11.3)	60 (11.5)	0.894
HR ≥ 100 bpm, n (%)	142 (13.5)	66 (12.9)	0.745
Killip classification			
I/ II/ III/ IV, n	721/166/62/103	332/76/42/67	0.071
Onset-to-door time ≥ 24 h, n (%)	109 (10.8)	46 (8.8)	0.223
Peak CPK level, mg/dL	1,884 (814–3,468)	1,951 (915–3,864)	0.630

Data are presented as n (%) or median (interquartile ranges). Values of CPK are the median (first to third quartile range). BP, blood pressure; BS, blood sugar; CABG, coronary artery bypass grafting; Cr: creatinine; Hb, hemoglobin; MI, myocardial infarction; PCI, percutaneous coronary intervention; WBC, white blood cell.

For each variable, the percentages reflect the total number of patients whose data are available.

Table 2 shows the lesion characteristics in the derivation and validation sets. The implementation of primary PCI showed no significant differences between the two sets.

**Table 2:** Lesion characteristics.

	Derivation set (n = 1,060)	Validation set (n = 521)	p value
CAG, n (%)	1,025 (96.7)	505 (96.9)	0.807
Primary PCI, n (%)	988 (93.2)	489 (93.9)	0.624
Culprit lesions			
LMT, n (%)	32 (3.1)	13 (2.6)	0.557
LAD, n (%)	517 (50.7)	260 (51.9)	0.657
RCA, n (%)	400 (39.2)	201 (40.1)	0.735
LCX, n (%)	111 (10.9)	45 (9.0)	0.251
Multi-vessel disease, n (%)	401 (39.0)	191 (36.7)	0.361
Pre TIMI 0/1/2/3, n	601/119/159/143	309/54/88/56	0.321
Post TIMI 0/1/ 2/3, n	22/13/48/941	12/9/36/449	0.196
Support device, n (%)	354 (26.6)	181 (28.0)	0.419
Temporary pacing, n (%)	127 (12.0)	71 (13.8)	0.327
IABP, n (%)	163 (15.4)	81 (15.7)	0.887
Respirator, n (%)	90 (8.5)	47 (9.1)	0.695
CHDF, n (%)	26 (2.5)	10 (1.9)	0.516

Data are presented as n (%). CAG, coronary angiography, CHDF, continuous hemodiafiltration; CPK, creatinine phosphokinase; IABP, intra-aortic balloon pumping; LAD, left anterior descending branch; LCX, left circumflex branch; LMT, left main trunk; RCA, right coronary artery; TIMI, thrombolysis in myocardial infarction. For each variable, the percentages reflect the total number of patients whose data are available.

Primary PCI was performed in nearly all cases (>95%) for STEMI patients. Infarct-related coronary artery and multi-vessel disease were similar between the two sets. We found that approximately 60% of cases had TIMI 0 flow in the two sets. TIMI classification and the use of supporting devices also showed no significant differences between the two sets. Moreover, similar population distribution was found between the two sets.

### Clinical outcomes in the derivation and validation sets

Table 3 shows the clinical outcomes during hospitalization. No significant difference was found in the in-hospital mortality rate with 10.8% and 11.7% in the derivation and validation sets, respectively. In addition, the rate of complications after primary PCI (cardiac rupture, subacute thrombosis, refractory VT/VF, and stroke) showed no significant differences between the two sets. The clinical outcomes including the in-hospital mortality and complications after primary PCI showed no significant differences between the two sets.

**Table 3:** Mortality and outcomes.

	Derivation set (n = 1,060)	Validation set (n = 521)	p value
In-hospital mortality, n (%)	114 (10.8)	61 (11.7)	0.579
Cardiogenic shock, n (%)	56 (5.3)	35 (6.7)	0.250
Cardiac rupture, n (%)	12 (1.1)	8 (1.5)	0.500
MOF, n (%)	20 (1.9)	6 (1.2)	0.280
CVA, n (%)	4 (0.4)	1 (0.2)	0.537
Renal failure, n (%)	6 (0.6)	4 (0.8)	0.634
Others, n (%)	24 (2.3)	10 (1.9)	0.657
Complications, n (%)	61 (6.1)	24 (4.6)	0.236
Cardiac rupture, n (%)	12 (1.1)	8 (1.5)	0.500
Subacute thrombosis, n (%)	20 (2.0)	3 (0.6)	0.031
Refractory VT/VF, n (%)	20 (2.0)	5 (1.0)	0.132
Stroke, n (%)	8 (0.1)	6 (1.2)	0.491

Data are presented as n (%). CVA, cerebrovascular attack; MOF, multiple organ failure; VT/VF, ventricular tachycardia/ventricular fibrillation. For each variable, the percentages reflect the total number of patients whose data are available.

### Multivariate analysis of laboratory variables

**Fig 2:**
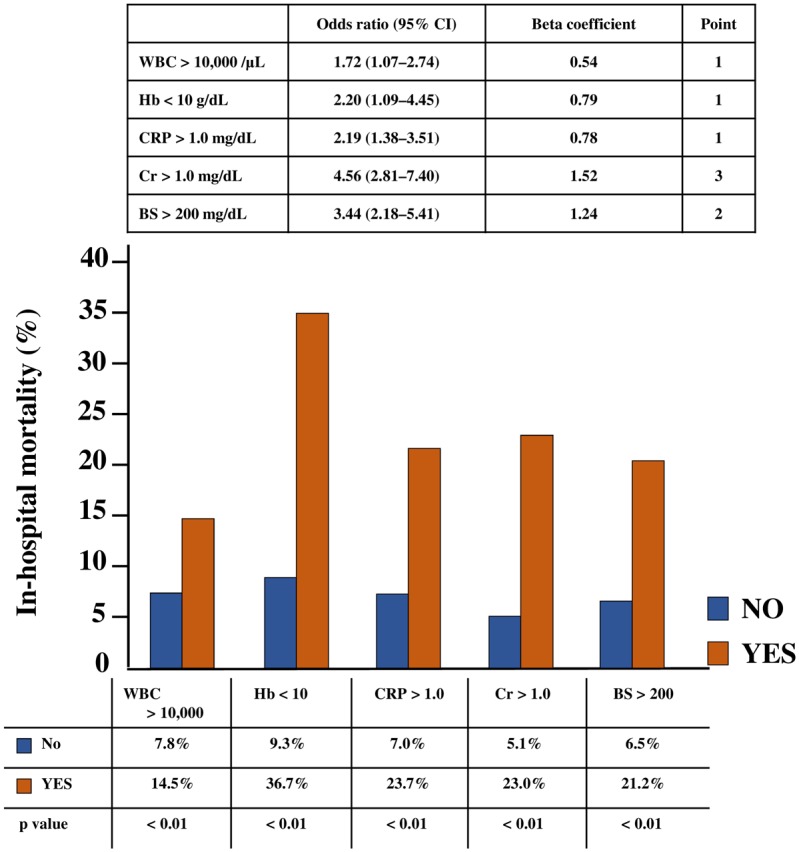
In-hospital mortality rate and multivariate analysis of laboratory variables in the derivation set.

WBC, anemia, CRP, renal function, and hyperglycemia have been reported as independent prognostic factors for AMI. We assessed the predictive power of these laboratory variables for the in-hospital STEMI mortality rate in the derivation set. Each variable (WBC > 10,000/μL, Hb < 10 g/dL, CRP > 1.0 mg/dL, Cr > 1.0 mg/dL, and BS > 200 mg/dL) was also associated with a significantly high in-hospital mortality rate in the derivation set (Fig 2). Based on the multivariate analysis for age ≥ 75 years, male sex, and these five laboratory variables, these variables remained as independent predictors for in-hospital mortality of STEMI in our derivation set; odds ratio (95% CI) was 1.72 (1.07–2.74), 2.20 (1.09–4.45), 2.19 (1.38–3.51), 4.56 (2.81–7.40), and 3.44 (2.18–5.41) for WBC, Hb, CRP, Cr, and BS, respectively (Fig 2).

### Risk stratification model of laboratory variables

**Fig 3:**
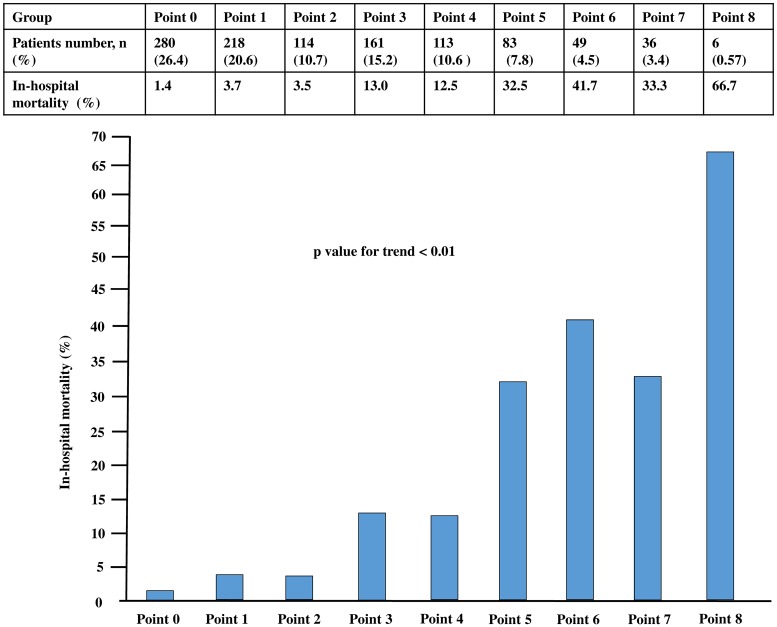
Risk point groups for predicting mortality in the derivation set.

**Fig 4:**
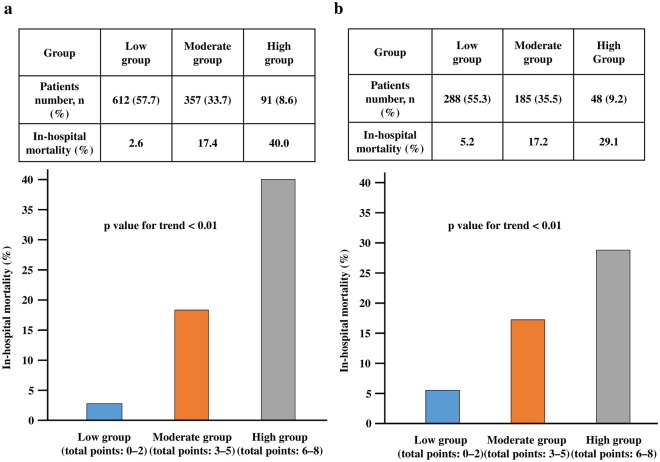
Risk of in-hospital mortality for laboratory risk groups. (a) Risk of in-hospital mortality in the derivation set. (b) Risk of in-hospital mortality in the validation set. Low-, moderate-, and high-risk groups have laboratory points from 0 to 2, 3 to 5, and 6 to 8, respectively.

**Fig 5:**
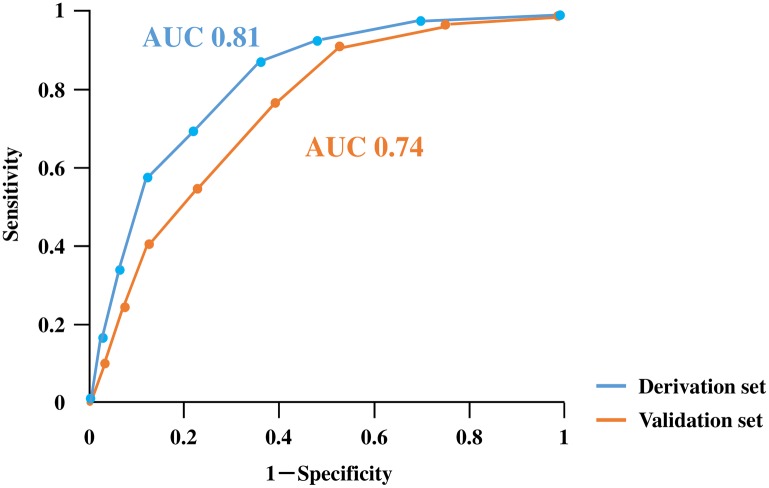
ROC curve of the derivation and validation set in the laboratory model. AUC, area under the curve.

To assess and triage the in-hospital mortality for STEMI simply and rapidly, we developed a risk stratification model from the combination of laboratory variables. We decided to assign points to each factor based on the beta coefficient of WBC and created a model that calculates the total points in the derivation set. Hb < 10 g/dL and CRP > 1.0 mg/dL are assigned 1 point because of the approximate beta coefficient of WBC > 10,000 /μL; Cr > 1.0 mg/dL is assigned 3 points because of the three-fold beta coefficient of WBC > 10,000 /μL; and BS > 200 mg/dL is assigned 2 points because of the two-fold beta coefficient of WBC > 10,000/μL (Fig 2). A significant trend in the in-hospital mortality rate was found for the total points of laboratory variables (Fig 3). We divided three risk groups based on the total points of the laboratory variables: low, moderate, and high laboratory groups corresponding to 0 to 2, 3 to 5, and 6 to 8 points, respectively. We also found a significant trend in the in-hospital mortality rate for the risk groups (low laboratory group, 2.6%; moderate laboratory group, 17.4%; and high laboratory group, 40.0%; p < 0.01) (Fig 4a). Therefore, we assessed the use of this risk stratification model in our validation set. In the validation set, each laboratory group had a significant trend for in-hospital mortality (low laboratory group, 5.2%, moderate laboratory group, 17.2%; high laboratory group, 29.1%; p < 0.01) (Fig 4b). In addition, we created ROC curves for our laboratory model in both the derivation and validation sets. The prognostic discriminatory capacity of our laboratory model was comparable to that of the full multivariable model (c statistic: derivation set vs validation set, 0.81 vs 0.74). Sorting patients into the risk groups based on the total points of laboratory variables was found to be a good element that could predict the in-hospital mortality of STEMI (Fig 5).

### Analysis of the laboratory risk stratification model combined with TIMI risk index

**Fig 6:**
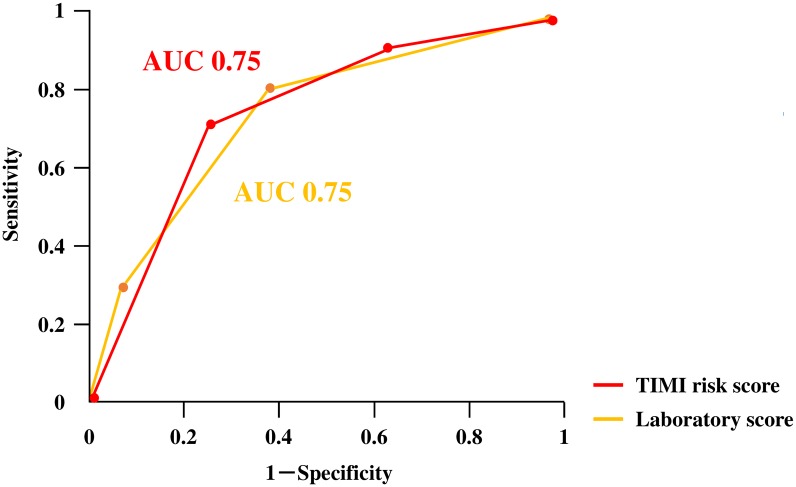
ROC curve of the laboratory model and the TIMI risk index in all STEMI cases. AUC, area under the curve.

**Fig 7:**
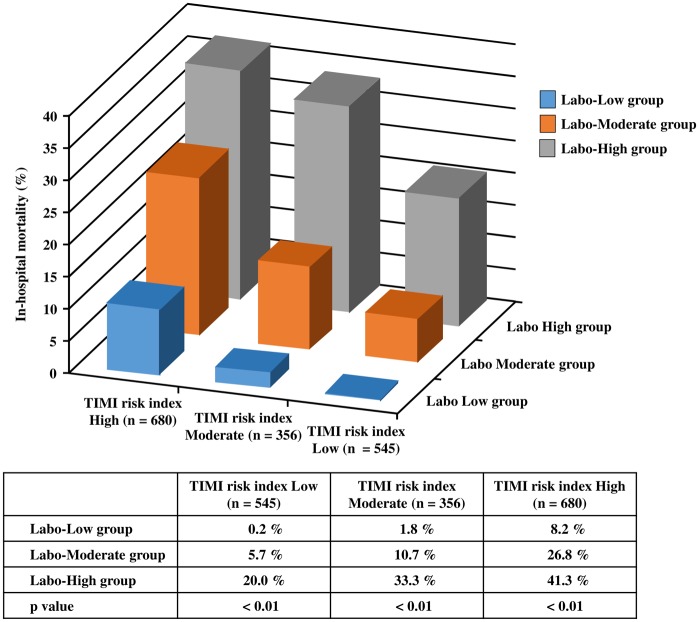
Subdivision by the laboratory risk in patients assessed with the TIMI risk index.

To further evaluate the laboratory risk stratification model, we investigated whether this model could be useful for the subdivision of the TIMI risk index. We calculated the TIMI risk index in all registered patients (n = 1,581) by using the three values reported as predictors of STEMI in an InTIME II substudy (age, SBP, and HR upon arrival)^3^. We divided the TIMI risk index into three groups based on the mortality rate in the InTIME II substudy. Cases with less than 22.5, from 22.5 to less than 30, and 30 or more points were classified as low, moderate, and high TIMI risk index groups, respectively. We created ROC curves for our laboratory model and TIMI risk index in all cases. The prognostic discriminatory capacity of our laboratory model was comparable to that of the full multivariable model (c statistic: the laboratory stratification model vs TIMI risk index, 0.75 vs 0.75). This result shows that the performance of our laboratory model is comparable to that of the TIMI risk index (Fig 6). The three TIMI risk index groups were further subdivided based on the laboratory risk stratification model, and in-hospital mortality rates in each laboratory risk group were examined. As shown in Fig 7, our laboratory risk stratification model combined with TIMI risk index at admission could predict the in-hospital mortality of STEMI (p < 0.01) simply and accurately compared with TIMI risk index alone. These results suggest that this risk stratification model from the combination of laboratory variables could become a simple and easy risk evaluation model (Fig 7).

## Discussion

The results of this registry showed that each of the five factors (WBC > 10,000/μL, Hb < 10 g/dL, CRP > 1.0 mg/dL, Cr > 1.0 mg/dL, and BS > 200 mg/dL) in the blood examination worsened in-hospital STEMI prognosis. In addition, the combination of these five factors could also predict the in-hospital mortality of STEMI. This laboratory risk stratification model combined with the TIMI risk index could be helpful in predicting the in-hospital STEMI prognosis in detail.

Few studies assessed the combination of laboratory variables for AMI prognosis. Patients with chronic kidney disease (CKD) were reported to have a significantly higher in-hospital mortality rate than the patients without CKD. Renal failure is also reported to be a cause for cardiovascular events, such as heart failure due to left ventricular insufficiency [10, 11, 15–17]. WBC and CRP are indicators of systemic inflammatory response activity. These inflammatory cytokines cause the promotion of infarction and remodeling of the left ventricle and increase the occurrence of cardiovascular events, including heart failure [8, 9, 18, 19]. Hyperglycemia also worsens in-hospital AMI prognosis whether diabetes mellitus is present or not. Hyperglycemia increases the incidence of peripheral arterial embolism during percutaneous treatment, infarction size, and acute kidney failure [12–14, 20, 21]. Anemia-induced tachycardia and low blood pressure result in the increase in AMI mortality rate. Studies have reported that cardiovascular events increase if Hb level decreases to less than 14 g/dL in STEMI patients, or the likelihood of cardiovascular death, myocardial infarction, and recurrent ischemia increases as the Hb level decreases to less than 11 g/dL in NSTEMI patients [10, 22].

In this study, each of the five factors was also verified to be significant in-hospital prognostic variables of STEMI. Combination of these laboratory variables could also be a risk model to predict the in-hospital STEMI prognosis. Several risk stratification models have been reported in the literature. The TIMI risk index is defined by age, SBP, and HR [4]. This risk index is used for assessment of severity for AMI and a triage during transportation. Although the TIMI risk index is useful for the patient triage, the SBP and HR during hospital transportation significantly fluctuate, especially in cases of profound tachycardia due to atrial fibrillation or endogenous catecholamine, and might be insufficient as prognostic factors in the acute phase. The TIMI risk score is based on previous medical history, examination findings, and medical interview [3]. Therefore, making an assessment is often difficult for serious cases or cases wherein data collections are inadequate. The Global Registry of Acute Coronary Events score [23] includes retrospective factors, such as whether the patient has had primary PCI; therefore, this score cannot be determined at admission. Our laboratory risk stratification model is simple and can assess the in-hospital STEMI prognosis at the primary stage. In addition, this laboratory model can be obtained from biochemical blood examination, which is routinely performed in cases of STEMI in clinical practice. This laboratory model assessed in-hospital mortality and can be used in determining treatment direction and triage in the acute phase of STEMI. Moreover, this model could further stratify the in-hospital STEMI prognosis by concurrently using the TIMI risk index. For these reasons, our laboratory model for STEMI could be useful in risk stratification for clinical use. (S2 Fig)

## Limitations

This study has several study limitations. First, this study is an observational analysis of a relatively small number of patients. Second, data regarding clinical background characteristics and angiographic results of primary PCI were unavailable for all study participants. We did not have appropriate detailed data regarding left ventricular function, ischemic time, and in-hospital medical treatments, which might be predictors for the in-hospital mortality of STEMI. In addition, the cutoff values for each of the five variables were determined by prioritizing figures within the spline curve created using the hospital prognosis with figures that can be used easily and conveniently in clinical practice. We could not obtain the data regarding the background wherein primary PCI was not performed. However, more than 90% of the subjects underwent primary PCI, and more than 90% were able to obtain TIMI III grade coronary flow as the TIMI classification after primary PCI, which is similar to actual clinical conditions. Despite these limitations, this laboratory risk stratification model could be useful for the evaluation of the STEMI prognosis.

## Conclusion

This laboratory stratification model could predict in-hospital mortality of STEMI simply and rapidly. This model could be useful for the detailed prediction of in-hospital STEMI mortality rate by further subdividing the TIMI risk index.

## Supporting Information

S1 Fig Spline curve for the cutoff values of five laboratory variables. WBC, White blood cell; Hb, Hemoglobin; CRP, C-reactive protein; Cr, Creatinine; BS, Blood sugar level.
(TIF)

S2 Fig Laboratory Risk Stratification Model for STEMI summarized for clinical use. WBC, White blood cell; Hb, Hemoglobin; CRP, C-reactive protein; Cr, Creatinine; BS, Blood sugar level.
(TIF)

S1 Table Ethics committees participated in this study. (DOCX)
