# Supplementary figures and images for: A Simple Risk Stratification Model for ST-Elevation Myocardial Infarction (STEMI) from the Combination of Blood Examination Variables: Acute Myocardial Infarction-Kyoto Multi-Center Risk Study Group
Source: PLoS One. 2016 Nov 11;11(11):e0166391. doi: 10.1371/journal.pone.0166391 (PMC5105954; doi:10.1371/journal.pone.0166391)

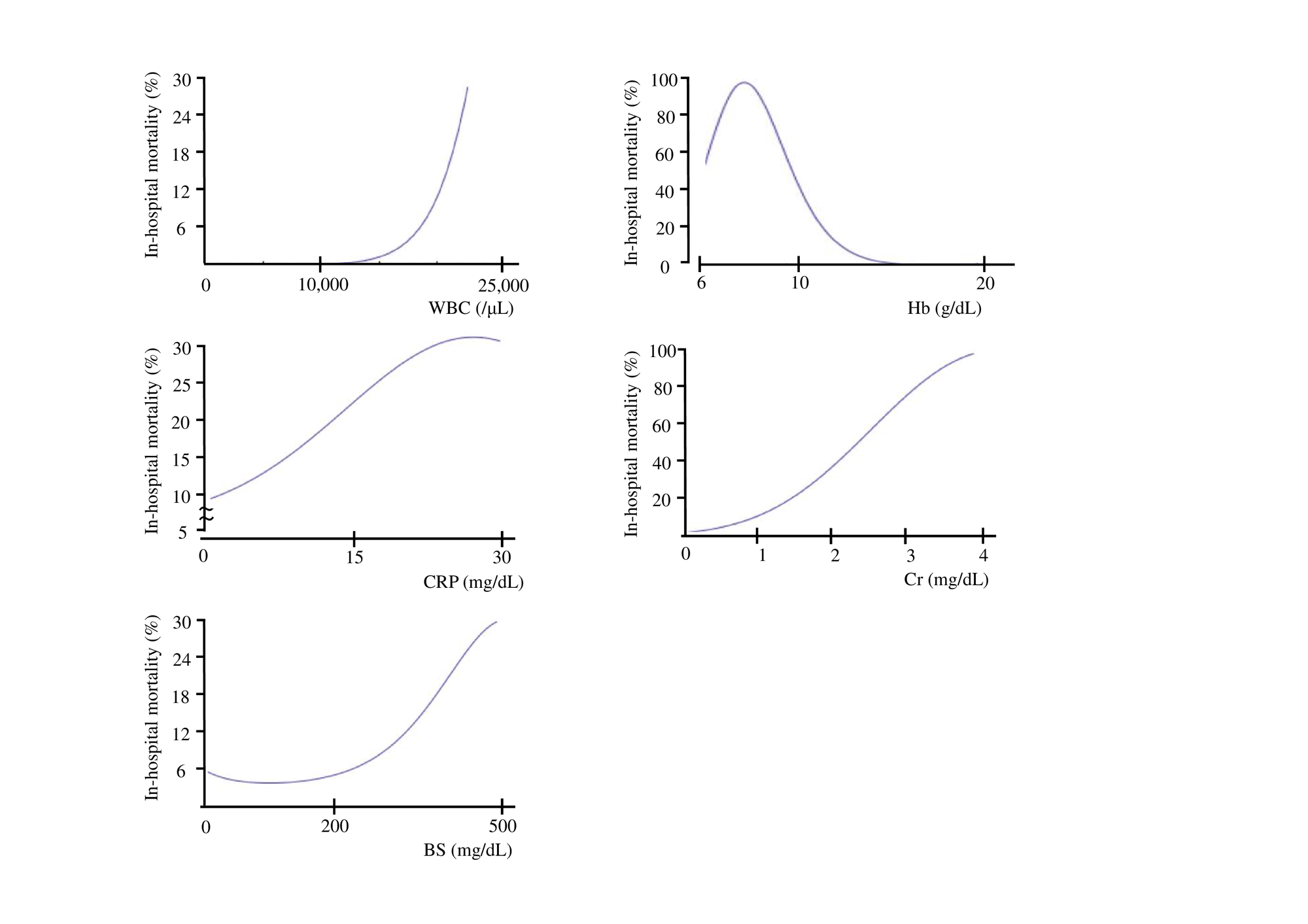

Supplement: S1 Fig — WBC, White blood cell; Hb, Hemoglobin; CRP, C-reactive protein; Cr, Creatinine; BS, Blood sugar level. (TIF) [file pone.0166391.s001.tif]

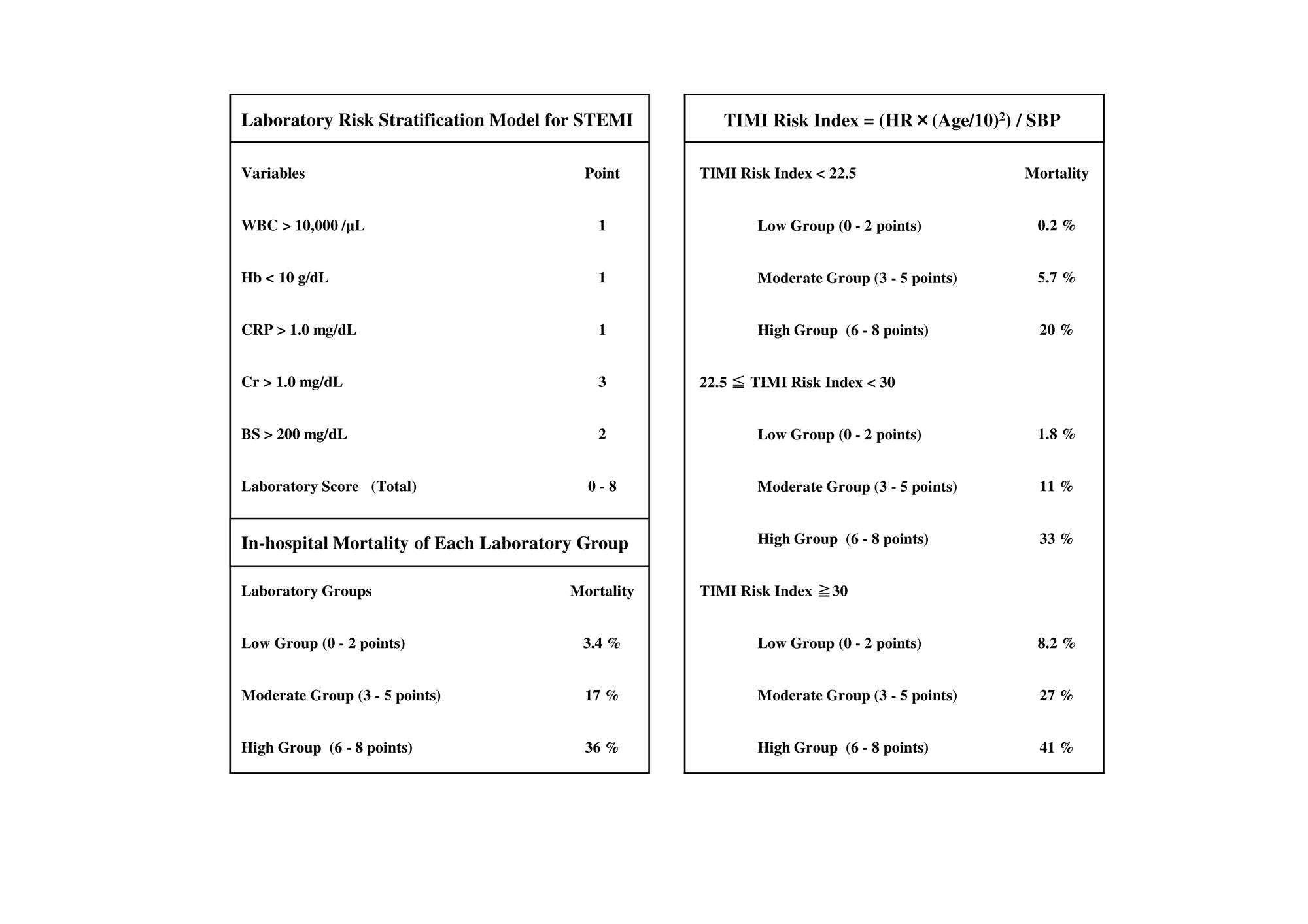

Supplement: S2 Fig — WBC, White blood cell; Hb, Hemoglobin; CRP, C-reactive protein; Cr, Creatinine; BS, Blood sugar level. (TIF) [file pone.0166391.s002.tif]
