# Supplementary material for: A Simple Risk Stratification Model for ST-Elevation Myocardial Infarction (STEMI) from the Combination of Blood Examination Variables: Acute Myocardial Infarction-Kyoto Multi-Center Risk Study Group
Source: PLoS One. 2016 Nov 11;11(11):e0166391. doi: 10.1371/journal.pone.0166391 (PMC5105954; doi:10.1371/journal.pone.0166391)
Supplement: S1 Table — (DOCX) [file pone.0166391.s003.docx]

| 1. Kyoto Prefectural University of Medicine |
| --- |
| 2. North Medical Center Kyoto Prefectural University of Medicine |
| 3. Kyoto First Red Cross Hospital |
| 4. Kyoto Second Red Cross Hospital |
| 5. Tanabe Central Hospital |
| 6. Kyoto City Hospital |
| 7. Nantan General Hospital |
| 8. Saiseikai Kyoto Hospital |
| 9. Saiseikai Siga Hospital |
| 10. Kouseikai Takeda Hospital |
| 11. Ayabe City Hospital |
| 12. Fukuchiyama City Hospital |
| 13. Kyoto Kizugawa Hospital |
| 14. Kyoto Yamashiro General Medical Center |
| 15. Ohmihachiman Community Medical Center |

**S1 Table. Ethics committees participated in this study.**
